# ASSOCIATION BETWEEN EXPOSURE TO HURRICANE HARVEY AND MORTALITY AMONG NURSING HOME RESIDENTS

**DOI:** 10.1093/geroni/igac059.1377

**Published:** 2022-12-20

**Authors:** Cassandra Hua, Kali Thomas, Sweta Patel, David Dosa, Dylan Jester, Ross Andel

**Affiliations:** Brown University School of Public Health, Providence, Rhode Island, United States; Brown University, Providence, Rhode Island, United States; Brown University, Providence, Rhode Island, United States; Brown University, Providence, Rhode Island, United States; University of California San Diego, La Jolla, California, United States; University of South Florida, Tampa, Florida, United States

## Abstract

Hurricane Harvey made landfall in Southeast Texas in August 2017, causing catastrophic flooding. We examined the association between exposure to Hurricane Harvey and rates of 30-and 90-day mortality among Texas nursing home residents. Using Medicare data, we compared a cohort of residents exposed to the storm in 2017 (n= 18,693) to a cohort of residents not exposed to the storm in 2015 (n=19,688). We fit generalized estimating equations with a sandwich estimator, adjusting for resident demographic and clinical characteristics. Exposure to Hurricane Harvey was not associated with 30-day rates of mortality. However, it was associated with a 15% increase in the odds of dying at 90 days holding resident characteristics constant (adjusted odds ratio: 1.15 [95% Confidence interval: 1.06, 1.25). Health effects due to severe flooding, such as those related to decreased ability to manage chronic conditions, may not be apparent until several weeks after the storm.

